# A novel function of FAF1, which induces dopaminergic neuronal death through cell-to-cell transmission

**DOI:** 10.1186/s12964-020-00632-8

**Published:** 2020-08-24

**Authors:** Gyeongrin Park, Bok-Seok Kim, Eunhee Kim

**Affiliations:** Department of Biological Sciences, Chungnam National University, 99 Daehak-ro, Yuseong-gu, Daejeon, 34134 South Korea

**Keywords:** FAF1, Secretion, Exosome, Vesicle-free form, Cell-to-cell transmission, Cell death

## Abstract

**Background:**

Fas-associated factor 1 (FAF1) has been implicated in Parkinson’s disease (PD) and activates the cell death machinery in the cytosol. However, the presence of extracellular FAF1 has not been studied.

**Methods:**

Serum-free conditioned medium (CM) from FAF1-transfected SH-SY5Y cells was concentrated and analyzed by western blotting. Exosomes were isolated from CM by ultracentrifugation and analyzed by western blotting, electron microscopy and nanoparticle tracking analysis. Soluble FAF1 from CM was immunodepleted using anti-FAF1 antibody. Transmission of secreted FAF1 was examined by transwell assay under a confocal microscope. CM-induced cell death was determined by measuring propidium iodide (PI) uptake using a flow cytometer.

**Results:**

FAF1 was secreted from SH-SY5Y cells via exocytosis and brefeldin A (BFA)-resistant secretory pathways. Furthermore, FAF1 was secreted as a vesicle-free form and a genuine exosome cargo in the lumen of exosomes. In addition, FAF1 increased the number of exosomes, suggesting a regulatory role in exosome biogenesis. Extracellular FAF1 was transmitted via endocytosis to neighboring cells, where it induced cell death through apoptotic and necrotic pathways.

**Conclusions:**

This study presents a novel route by which FAF1 induces neuronal death through cell-to-cell transmission.

Video Abstract

## Background

Cells secrete proteins harboring signal peptides through the classical secretory pathway via the endoplasmic reticulum (ER)-Golgi complex, from which vesicles with cargo proteins move toward and fuse with the plasma membrane and subsequently export cargos to the extracellular space [1]. However, proteins lacking signal peptides are secreted via alternative, nonclassical secretory pathways [2]. These pathways are classified as vesicular and nonvesicular secretory pathways [3]. Some proteins are secreted via extracellular vesicles including exosomes and other vesicles of various sizes [4, 5]. Alternatively, other proteins are secreted through membrane pores and ATP-binding cassette (ABC) transporters, although the exact mechanisms of nonvesicular secretory pathways are elusive [6, 7].

Exosomes, which are nanosized membrane vesicles (50–150 nm in diameter) secreted into the extracellular environment by various cell types, are associated with intercellular communication with neighboring cells and play a role in a myriad of pathological functions in diseases including cancer, cardiovascular, and neurodegenerative diseases [8–13]. In particular, exosomes remodel the extracellular matrix and promote metastasis, angiogenesis, thrombosis, and tumor cell proliferation in cancer [14]. Exosomes in cardiovascular disease display proangiogenesis, procoagulant, and proinflammatory effects on the vessel walls [15]. In neurodegenerative diseases, exosomes are potential sources of key pathogenic proteins such as tau, β-amyloid, prion, and α-synuclein [16–19].

In addition to their vesicle-mediated secretion, many proteins are secreted through nonvesicular mechanisms. For instance, the proteins such as tau and fibroblast growth factor 2 are recruited to the plasma membrane to form lipidic pores and are subsequently secreted [7, 20]. In addition, inflammation triggers the secretion of interleukin-1β and transglutaminase 2 through pores [21]. Fibroblast growth factor 1 translocates across the plasma membrane via poorly understood mechanisms, including the membrane release complex, upon stress [22]. Proteins such as hydrophilic acylated surface protein B are secreted by ABC transporters [23]. Proteins secreted via nonvesicular secretory pathways are advantageous over cargo proteins in vesicles as immunotherapeutic targets because of the antibody accessibility of the extracellular space.

Fas-associated factor 1 (FAF1) is involved in diverse biochemical processes including cell death, inflammation, cell proliferation, and proteostasis [24–28]. Consistent with its diverse functions, FAF1 has been implicated in certain diseases [29]. First, FAF1 plays a tumor- suppressive role through activation of the apoptotic machinery and NF-κB suppression [25, 26, 30]. FAF1 also suppresses tumor metastasis via TGF-β signaling. Moreover, FAF1 expression is downregulated in various cancers, including lung, colon, liver, prostate, brain, ovarian, and breast cancers [31]. Second, FAF1 is involved in Parkinson’s disease (PD) as it is overexpressed in PD patients, colocalizes with α-synuclein, and acts as a substrate of parkin. Furthermore, FAF1 mediates the degeneration of dopaminergic neurons through apoptosis and parthanatos [32–34].

FAF1 is an intracellular protein present mainly in the cytosol. To date, FAF1 secretion has not yet been reported. Herein, we uncovered for the first time that FAF1 is secreted via an ER/Golgi-independent pathway. Specifically, FAF1 is secreted through exosomal and nonvesicular pathways in SH-SY5Y cells. In addition, FAF1 augments the number of exosomes, suggesting the involvement of FAF1 in exosome biogenesis. Furthermore, extracellular FAF1 moves into neighboring cells via pinocytosis and clathrin-mediated endocytosis. Transmitted FAF1 induces cell death via apoptosis and necrosis. Collectively, these results present a novel measure by which FAF1 propagates its death function through cell-to-cell transmission.

## Methods

### Reagents and antibodies

The following reagents and antibodies used in this study were purchased commercially: TNFα from AbFrontier (Seoul, South Korea); z-IETD-fmk (caspase-8 inhibitor), mouse anti-HA antibody and rabbit anti-GM130 antibody from Abcam (Cambridge, UK); 2′,7′-dichlorofluorescin diacetate (DCFH-DA), hydrogen peroxide (H_2_O_2_), 1-methyl-4-phenylpyridinium (MPP^+^), propidium iodide (PI), poly-D-lysine, brefeldin A (BFA), GW4869, monensin, cycloheximide (CHX), necrostatin-1 (Nec-1), DPQ, proteinase K (PK), Dynasore, heparin, Heparinase III, mouse anti-β-actin, and mouse anti-Flag antibody from Sigma-Aldrich (Saint Louis, MO, USA); 4′,6-diamidino-2-phenylindole (DAPI), horseradish peroxidase (HRP)-conjugated anti-mouse antibody, and HRP-conjugated anti-rabbit antibody from Thermo Fisher Scientific, Inc. (Rockford, IL, USA); mouse anti-Flotillin-1 from BD Biosciences (San Jose, CA, USA); bafilomycin A1, mouse anti-Alix antibody, mouse anti-β-Galactosidase antibody (40-1a), rabbit anti-Calregulin antibody, mouse anti-CD63 antibody, mouse anti-Parkin antibody, and mouse anti-FAF1 antibody from Santa Cruz Biotechnology (Dallas, TX, USA); mouse anti-Hsc70 antibody and mouse anti-Hsp90 antibody from Enzo Life Sciences (Farmingdale, NY, USA); And zVAD-fmk (z-VAD) from Calbiochem (Darmstadt, Germany).

### Cell culture and transfection

SH-SY5Y, MEF, HEK293, RAW264.7, HeLa, PANC-1, MIA PaCa-2 and MCF-7 cells were maintained in Dulbecco’s modified Eagle’s medium (DMEM, WelGENE, Daegu, Korea) containing 10% fetal bovine serum (FBS, Atlas Biologicals, Fort Collins, CO, USA) and 1% antibiotic-antimycotic (Gibco BRL, Grand Island, NE, USA) unless otherwise specified. Cells were transfected with the indicated plasmids using Bio T (Morganville Scientific Inc., Morganville, NJ, USA) following the manufacturer’s protocol. Rat midbrain cultures derived from postnatal day 1 were prepared using standard procedures. Briefly, material dissected form the ventral portion of the midbrain was cleaned free of meningeal tissue, minced, and enzymatically dissociated in a mixture of papain/DNase (Sigma-Aldrich). Dissociated cells were plated onto amine-coated 6-well plates (BD Biosciences). Cells were maintained in neurobasal medium (Gibco), with B27 serum-free supplements (Gibco), 0.5 mm l-glutamine. After 5 days of culture, the cells were infected using AAV1 (adeno-associated virus 1)-hFAF1 viral vectors (MOI of 10,000 and 50,000).

### Site-directed mutagenesis

The siRNA-resistant parkin construct was generated using QuikChange site-directed mutagenesis kit (Stratagene, La Jolla, CA, USA). The primers were as follows: #1; 5′-GAGCTGAGAAACGACTGGACTGTGCAGAATTGTG-3′, #2; 5′-GGGAAGGAGCTGAG AAACGATTGCACTGTGCAGA -3′.

### RNA interference

All small interfering RNAs (siRNAs) against parkin and scrambled RNA (scRNA) were purchased from Bioneer (Daejeon, South Korea). The sequences of the siRNAs used in this study were as follows: siRNA against parkin (5′-UGAGGAAUGGGACUGU-3′). The scRNA or siRNA were transfected into SH-SY5Y cells using Lipofectamine RNAi MAX (Thermo Fisher Scientific) according to the manufacturer’s instructions.

### Preparation of conditioned medium

To prepare conditioned medium (CM), cells transfected with the indicated plasmids were cultured in 60 mm diameter dishes in DMEM containing 10% FBS. After 24 h, the cells were switched to serum-free DMEM for the indicated times. Here, we used serum-free medium to avoid interference from albumin-enriched FBS. Then, the CM was collected and centrifuged at 800⨯g for 5 min and 2000⨯g for 10 min to remove cellular debris. For western blot analysis, the CM was concentrated using 50 kDa or 100 kDa cutoff Amicon Ultra filters (Millipore, Billerica, MA, USA) at 4000⨯g for 10 min or 15 min.

### Western blot analysis

Cells were harvested, washed twice with PBS and lysed with mammalian lysis buffer [50 mM Tris-Cl (pH 8.0), 150 mM NaCl, 1 mM EDTA, 1% Nonidet P-40, 0.4 mM phenylmethylsulfonylfluoride]. Then, the protein concentrations were quantified by using a Bio-Rad protein assay kit (Bio-Rad, Hercules, CA, USA). After quantification, samples were boiled in 6× protein sample buffer [250 mM Tris-Cl (pH 6.8), 30% glycerol, 10% SDS, 5% β-mercaptoethanol]. Then, samples were electrophoresed by SDS-PAGE and transferred to nitrocellulose membranes (GE Healthcare, Maidstone, UK). The membranes were blocked with 5% skim milk in PBS with 0.1% Tween-20 (PBST) and incubated with the indicated primary antibodies overnight. After their washing with PBST, the membranes were incubated with secondary antibodies. Immunoblot signals were measured by using chemiluminescent detection (Lab Frontier, Anyang, Korea).

### CHX chase assay

SH-SY5Y cells were transiently transfected with the indicated plasmids for 24 h. After transfection, the cells were switched to serum-free DMEM containing CHX (Sigma-Aldrich, 20 μg/ml) for the indicated of times. Subsequently, the medium was concentrated with 50 kDa cutoff Amicon Ultra filters (Millipore) and analyzed by western blot analysis.

### Purification of exosomes

We followed a previously described protocol with some modifications [35]. Briefly, cells were transfected with the indicated plasmids for 24 h and incubated in DMEM containing 10% exosome-depleted FBS (System Biosciences, Palo Alto, CA, USA) for 48 h. The CM was then collected and subjected to sequential centrifugation at 800⨯g for 5 min, 2000⨯g for 10 min and 10,000⨯g for 30 min at 4 °C to remove cellular debris. Using a Beckman Coulter Optima L-90 K ultracentrifuge with a type 41Ti rotor, the supernatant was then spun down at 140,000⨯g for 70 min. The pellet was resuspended in PBS and then spun again at 140,000⨯g for 70 min at 4 °C. Finally, the pellet was resuspended in PBS or radioimmunoprecipitation assay (RIPA) buffer (Sigma-Aldrich).

In addition to their isolation via ultracentrifugation, we isolated exosomes using ExoQuick-TC (System Biosciences) according to the manufacturer’s instructions.

### Treatment of vesicles with Na_2_CO_3_

To separate integral membrane proteins and luminal proteins, purified exosomes were treated with 100 mM Na_2_CO_3_ (pH 11) for 30 min at 4 °C as previously described [36]. After centrifugation at 50,000⨯g for 60 min, integral proteins remained in the pellet fraction, while luminal proteins remained in the supernatant fraction. The pellet fractions were resuspended in RIPA buffer (Sigma-Aldrich) and the supernatants were collected in a separate tube for western blot analysis.

### Proteinase K digestion

PK (Sigma-Aldrich) was added to the samples at a final concentration of 2 μg/ml. Then, the samples were incubated at 37 °C for 30 min and 5 mM phenylmethylsulfonyl fluoride was added to inhibit the activation of PK, followed by the addition of protein sample buffer.

### Immunodepletion of CM

Fifty microliters of protein G-conjugated Dynabeads (ThermoFisher Scientific) was incubated overnight with mouse monoclonal antibody against FAF1 (final concentration of 0.5 or 1 μg/ml) before its addition to CM. The antibody-Dynabeads complex was incubated with 3 ml of CM at 4 °C overnight. After the complex was removed using a magnet, the immunodepleted CM was concentrated using a 50 kDa cutoff Amicon Ultra filter and used for western blot analysis. For flow cytometry, unconcentrated immunodepleted CM was applied to recipient cells.

### Flow cytometry

To evaluate cell death, we measured PI-positive cell staining by using a Guava EasyCyte flow cytometer (Millipore). Briefly, cells were switched to serum-free DMEM or neurobasal medium for the indicated time. Additionally, to measure recipient cell death, SH-SY5Y and rat primary neuronal cells were treated with CM from donor cells for the indicated times. The cells were harvested, stained with PI (50 μg/ml), and evaluated using a Guava EasyCyte flow cytometer, following which the results were quantified using InCyte software (Millipore).

### Concentrated CM treatment

SH-SY5Y cells (donor cells) were plated on 60 mm tissue culture dishes and transfected with GFP-vector or GFP-FAF1 plasmid. At 24 h after transfection, the cells were incubated in serum-free medium for 24 h. After the CM was concentrated by using 50 kDa cutoff Amicon Ultra filters, the concentrated CM was dissolved in new serum-free medium that was applied to recipient cells on poly-L-lysine-coated coverslips in 12-well plates for 24 h.

### Propagation assay

SH-SY5Y cells (donor cells) were plated on 60 mm tissue culture dishes. Donor cells were transfected with GFP-vector or GFP-FAF1 plasmids. At 24 h after transfection, the cells were collected and replated in cell culture inserts (polycarbonate membrane, 3.0 μm pore size, Corning, Kennebunk, ME, USA) at a density of 1⨯10^5^ cells. SH-SY5Y cells (recipient cells) were plated at a density of 1⨯10^5^ cells on poly-D-lysine-coated coverslips in 12-well plates. After 24 h, the cultures were combined such that the donor cells were in the insert and separated from recipient cells plated on a coverslip.

### Confocal microscopy

Cells were fixed with 4% paraformaldehyde for 15 min. The *cis*-Golgi were stained with GM130. After the nuclei were stained with DAPI for 10 min. The coverslips were mounted onto microscope slides using fluorescence mounting medium (Dako, Carpinteria, CA, USA) and analyzed using a Zeiss LSM 510 laser scanning confocal microscope (Carl Zeiss, Oberkochen, Germany).

### Electron microscopy

For transmission electron microscopy (TEM), samples were prepared using the Exosome-TEM-easy Kit containing a Formvar-carbon-coated EM mesh 400 grid, wash buffer, and EM solution (101 Bio, Mountain View, CA, USA). The pellets from the 12 ml of CM (vector- or FAF1-transfected cells) obtained by ultracentrifugation were resuspended in 60 μl of PBS, 10 μl of which was applied to the grid. All samples were prepared following the manufacturer’s instructions. For immuno-EM, the pellets were first fixed with 40 μl of 4% paraformaldehyde and 0.2% glutaraldehyde (Sigma-Aldrich) overnight at 4 °C. Then, the fixed exosome solution was transferred to grids and subsequently treated with 0.05 M glycine for 10 min to quench free aldehyde groups. After blocking with PBS containing 1% BSA for 30 min, the grids were incubated for 1 h with the indicated antibodies (diluted 1:50 in PBS containing 0.1% BSA) at room temperature. After three washes with PBS containing 0.1% BSA, the grids were incubated for 1 h with the secondary antibody (anti-mouse IgG conjugated to 10 nm gold particles, 1:25, Sigma-Aldrich) at room temperature. Three washes to eliminate secondary antibody were followed by incubation with EM solution and a wash step. Samples were viewed under a Talos F200X transmission electron microscope (FEI, Hillsboro, OR, USA) operated at 200 kV, and images were captured with a Ceta 16 M pixel CMOS camera (FEI).

### Nanoparticle tracking analysis

Following isolation by differential ultracentrifugation or ExoQuick-TC (System Biosciences), the exosome pellets were resuspended in 50 μl of PBS. Then, 10 μl of the exosome solution was diluted in PBS to a total volume of 1 ml. The samples were analyzed by nanoparticle tracking analysis using a NanoSight NS300 (Malvern Panalytical Ltd., Malvern, UK), equipped with a 405 nm laser. To accurately identify the vesicles, the detection threshold was set at 5. The number of vesicles in each sample represents the number of particles per ml of medium. Cells were counted using a Muse Count & Viability Kit (Millipore) on a Muse cell analyzer (Millipore).

### Caspase-3 activity assay

SH-SY5Y cells were treated with CM from FAF1-transfected cells plus caspase-8 inhibitor or TNFα (Ab frontier) plus CHX (Sigma-Aldrich) at the indicated concentrations for the indicated times. Then, caspase-3 activity was measured by using a caspase-3 colorimetric assay kit (BioVision, Milpitas, CA, USA) in accordance with the manufacturer’s protocol. The absorbance at 450 nm was measured with the use of a VICTOR microplate reader (PerkinElmer, Norwalk, CT, USA).

### SignalP-4.1

We investigated the presence of a signal peptide in FAF1 using SignalP-4.1 (http://www.cbs.dtu.dk/services/SignalP-4.1/) with secretogranin-1 used as a positive control as it contains a signal peptide.

### Statistical analyses

Experiments were independently carried out three times (*n* = 3). All the data are expressed as the mean ± standard deviation (S.D.). Statistical comparisons were performed using Student’s t-test or one-way analysis of variance (ANOVA) followed by Tukey’s HSD post hoc analysis using SPSS software (statistics version 22; IBM, Inc., Chicago, IL, USA). Statistical significance was established when the *P*-value was lower than 0.05.

## Results

### FAF1 is secreted via nonclassical exocytosis

According to the CSF Proteome Resource, FAF1 is detected in the cerebrospinal fluid and plasma (Additional file 1). This study aimed to determine whether FAF1 is also secreted at the cellular level and investigate FAF1 secretion in SH-SY5Y human neuroblastoma cells.

**Fig. 1:**
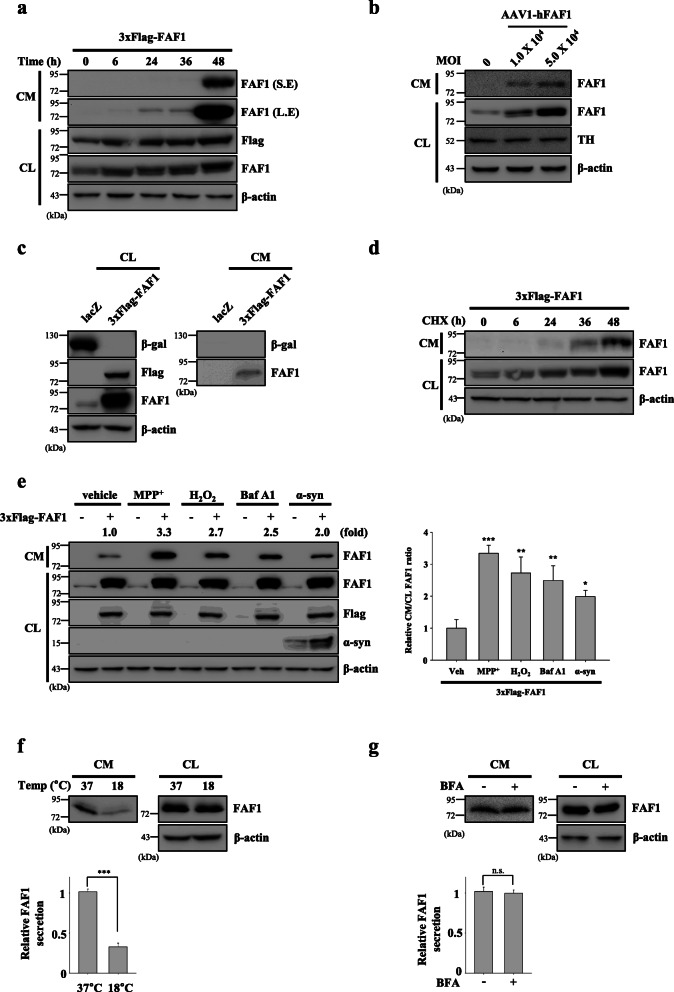
FAF1 is secreted via nonclassical exocytosis. **a** SH-SY5Y cells were transfected with vector control (VC) or 3xFlag-FAF1 plasmid. At 24 h after transfection, the culture medium was replaced with serum-free medium for the indicated times. S.E.: short exposure, L.E.: long exposure. **b** Rat primary neuronal cells were transduced with AAV1-hFAF1 viral vectors. At 3 days after transduction, the culture medium was replaced with serum-free neurobasal medium for 48 h. **c** Cells were transfected with lacZ or 3xFlag-FAF1 plasmid. At 24 h after transfection, the culture medium was replaced with serum-free medium, and cells were cultured for 24 h. **d** Cells were transfected with VC or 3xFlag-FAF1 plasmid. At 24 h after transfection, the culture medium was replaced with serum-free medium containing CHX (20 μg/ml) for the indicated times. **e** Left panel: Cells were transfected with VC or 3xFlag-FAF1 plus α-syn plasmid. At 24 h after transfection, the culture medium was replaced with serum-free medium containing DMSO (vehicle), MPP^+^ (1 mM), H_2_O_2_ (100 μM), or Baf A1 (50 nM) for 24 h. Right panel: The graph shows the densitometric analysis of immunoblotting of FAF1 in conditioned medium (CM) shown in the left panel (*n* = 3). **f** Upper panel: Cells were transfected with 3xFlag-FAF1 plasmid. At 24 h after transfection, the culture medium was replaced with serum-free medium, and the cells were cultured at 18 °C or 37 °C for 24 h. Lower panel: The graph shows the result of densitometric analysis of FAF1 immunoblotting in CM shown in the upper panel (*n* = 3). **g** Upper panel: Cells were transfected with 3xFlag-FAF1 plasmid. At 24 h after transfection, the culture medium was replaced with serum-free medium containing BFA (2 μg/ml) for 24 h. Lower panel: The graph shows the result of densitometric analysis of FAF1 immunoblotting in CM shown in the upper panel (*n* = 3). Cell lysate (CL) and concentrated CM were analyzed by western blotting with the indicated antibodies. All lanes were loaded with the same amount of total protein. The data are expressed as the mean ± S.D. of three independent experiments. Statistical comparisons were performed using using ANOVA followed by Tukey’s HSD post hoc analysis **(e)** and Student’s t-test (**f** and **g**). ^*^P < 0.05, ^**^P < 0.01, ^***^P < 0.001 and n.s. = not significant

Because FAF1 is a death-promoting protein [24], sublethal experimental FAF1 transfection conditions were used to exclude cellular debris due to death (Additional file 2: Fig. S1a and b). The FAF1 transfection conditions in SH-SY5Y cells under which FAF1 secretion, but not cell death, occurs were determined by PI staining followed by flow cytometry. Cells were transfected with 3xFlag-FAF1 plasmid at a sublethal dose for 24 h and subsequently moved to serum-free medium. FAF1 was detected in the serum-free medium at 6 h and accumulated henceforth, indicating that FAF1 is secreted in a time-dependent manner (Fig. 1a). This finding suggests that FAF1 is constitutively released from SH-SY5Y cells. To exclude a tagging artifact, another epitope tag, hemagglutinin (HA), was used. HA-FAF1 was also detected in the CM (conditioned medium) in a dose-dependent manner (Additional file 2: Fig. S1c). Furthermore, we examined the FAF1 secretion with rat primary neurons using AAV1 (adeno-associated virus 1)-mediated FAF1 overexpression. FAF1 overexpression in rat primary neuronal cells demonstrated consistent results with that in SH-SY5Y cells (Fig. 1b). Moreover, β-galactosidase was not detected in the CM of β-galactosidase-transfected cells, demonstrating that the release of FAF1 is not a transfection artifact (Fig. 1c) [37]. Next, a pulse-chase experiment using CHX to inhibit de novo protein synthesis was performed. Consistently, FAF1 was secreted and accumulated over time, further confirming that FAF1 is constitutively secreted from SH-SY5Y cells (Fig. 1d). Next, we examined whether FAF1 is also secreted by other cells. FAF1 was detected in the extracellular space of MEFs and HEK293 cells. Furthermore, FAF1 was present in the CM of each of a number of various cancer cell types (MCF-7, HeLa, PANC-1, and MIA PaCa-2 cells). This shows that FAF1 secretion is not specific to SH-SY5Y cells (Additional file 3: Fig. S2a-f).

Because FAF1 has been implicated in PD pathogenesis, FAF1-transfected SH-SY5Y cells were treated with the PD-associated stressors such as MPP^+^, H_2_O_2_, bafilomycin A1 and α-synuclein overexpression at sublethal doses (Additional file 4: Fig. S3). There were no significant changes of FAF1 expression in CLs (cell lysates) dependent on various PD-associated stressor types. However, FAF1 secretion increased in CMs upon all stressors used in this study, implying that PD-associated stressors positively regulate FAF1 secretion (Fig. 1e).

To elucidate the mechanism by which FAF1 is secreted, two sets of experiments were performed as follows. First, we examined whether FAF1 is released via exocytosis or passive diffusion. As exocytosis is affected by temperature [38], FAF1-transfected SH-SY5Y cells were incubated at either 18 °C or 37 °C. FAF1 secretion at 18 °C was significantly reduced compared to that at 37 °C, indicating that FAF1 is released via exocytosis (Fig. 1f). Second, we examined whether FAF1 is secreted via the classical ER/Golgi-dependent secretory pathway using BFA which generates ROS and disassembles Golgi through ER/Golgi pathway inhibition. BFA did not affect FAF1 release (Fig. 1g, Additional file 2: Fig. S1d) [39]. Furthermore, a signal peptide was not found in FAF1 when its sequence was analyzed using SingalP4.1, further excluding the possibility of ER/Golgi-mediated secretion of FAF1 (Additional file 5: Fig. S4). Taken together, these data demonstrate that FAF1 is released via nonclassical exocytosis.

### FAF1 is secreted via exosomal and nonvesicular pathways

**Fig. 2:**
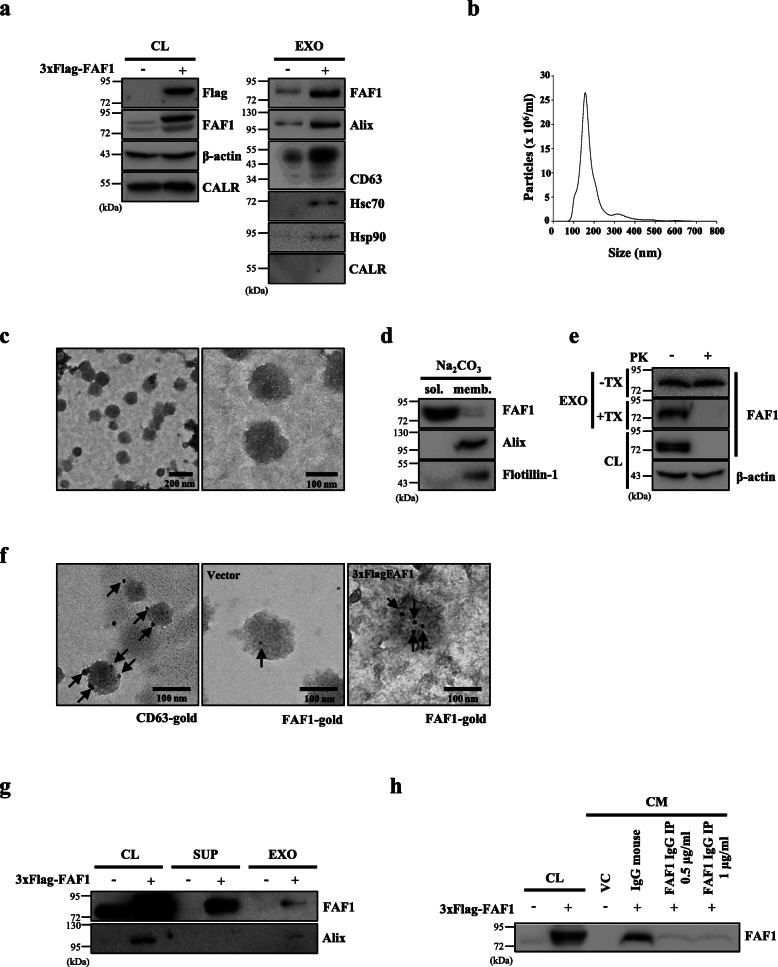
FAF1 is released to the extracellular space via both exosomal and nonvesicular pathways. **a** SH-SY5Y cells plated on 150 mm dishes were transfected with VC or 3xFlag-FAF1 plasmid. At 24 h after transfection, the culture medium was replaced with exosome-depleted medium, and the cells were cultured for 48 h. Then, exosomes were isolated from the CM by ultracentrifugation. CL and isolated exosomes (EXOs) were analyzed by western blotting with the indicated antibodies. CALR: calregulin. **b** Purified exosomes were characterized using nanoparticle tracking analysis. **c** Representative TEM images of exosomes are shown. Scale bar: 200 nm (left) or 100 nm (right). **d** The purified exosomes were treated with Na_2_CO_3_. After centrifugation at 50,000⨯g, the integral membrane proteins were pelleted (memb.), and nonintegral and luminal proteins remained in the supernatant (sol.). These fractions were analyzed by western blotting with the indicated antibodies. **e** The purified exosomes were incubated with PK (2 μg/ml) in the absence or presence of 1% Triton X-100 (TX). +Tx, with 1% TX; −Tx, without TX. **f** Immunogold labeling of purified exosomes with anti-CD63 antibodies (left) and anti-FAF1 antibodies from VC-transfected (middle) or 3xFlag-FAF1-transfected (right) cells. Scale bar: 100 nm. **g** Cells were transfected with VC or 3xFlag-FAF1 plasmid. At 24 h after transfection, the culture medium was replaced with serum-free medium, and the cells were cultured for 24 h. After the CM was isolated by ultracentrifugation, the supernatant (SUP) and pellet (EXO) were analyzed by western blot with the indicated antibodies. **h** After immunoprecipitation of FAF1 from CM with anti-FAF1 monoclonal antibody, the immunodepleted CM was concentrated and analyzed by western blotting with the indicated antibodies. All lanes were loaded with the same amount of total protein

To determine the mechanism by which FAF1 is secreted, exosomes were isolated from CM using a differential ultracentrifugation procedure as previously described [35] and ExoQuick-TC following the manufacturer’s protocol. Western blot analysis of exosomes isolated by ultracentrifugation revealed the presence of the exosome markers Alix, CD63, Hsc70, and Hsp90, but not calregulin, an ER resident protein 60 (a negative exosome control) (Fig. 2a). Exosomes isolated from SH-SY5Y cells by ExoQuick-TC showed an exosome marker profile consistent with that of exosomes purified by ultracentrifugation (Additional file 6: Fig. S5a). Moreover, nanoparticle tracking analysis and TEM data further confirmed that our purified exosomes exhibited a typical size distribution with a diameter ranging from 50 to 150 nm and a typical morphology of exosomes (Fig. 2b and c).

Endogenous as well as overexpressed FAF1 proteins were detected in the exosomal fractions isolated by both methods, indicating that FAF1 is secreted as a genuine exosomal cargo (Fig. 2a). Similarly, FAF1 was also found in exosomes isolated from the various indicated cell lines by ExoQuick-TC (Additional file 6: Fig. S5b-h). Next, we investigated whether FAF1 is present on the membrane or in the lumen of exosomes. Exosomes were treated with 100 mM Na_2_CO_3_ (pH 11) to distinguish between the exosomal membrane and the lumen. Both Alix and flotillin-1 were present in the exosomal membrane (positive controls), whereas FAF1 was mainly present in the lumen of the exosomes (Fig. 2d). The topology of exosomal FAF1 was further examined using PK, a nonspecific protease, to digest proteins outside of the exosomes. Exosomal FAF1, but not cytosolic FAF1, was protected from PK treatment in the absence of Triton X-100 (TX) (Fig. 2e, −TX). In contrast, PK treatment with TX disintegrating the exosome structures abolished the exosome-mediated protection of FAF1 (Fig. 2e, +TX). Our data demonstrated the presence of FAF1 in the lumen of exosomes. Furthermore, the presence of FAF1 in the exosomal lumen was confirmed with the visualization of immunogold-labeled FAF1 under an electron microscope. As shown in Fig. 2f, immunogold-labeled CD63 was mainly present outside exosomes, whereas immunogold-labeled FAF1 was present in the lumen of exosomes. Collectively, these results consistently showed that FAF1 is located in the lumen of exosomes.

Because certain exosomal cargo proteins are also secreted via the nonvesicular secretory pathway [18, 40–42], the possibility that FAF1 is also secreted via a nonvesicular route was investigated. To this end, the CM was fractionated into exosomal (pellet) and nonexosomal (supernatant) fractions by ultracentrifugation [40]. FAF1 from the CM was present in nonexosomal as well as exosomal fractions (Fig. 2g), implying the presence of a soluble form of FAF1. To further investigate this, CM from FAF1-transfected SH-SY5Y cells was treated with anti-FAF1 antibody. One microgram of anti-FAF1 antibody almost eliminated FAF1 from the CM, indicating that FAF1 is predominantly secreted in a soluble form (Fig. 2h). Taken together, these results demonstrate that FAF1 is concurrently released as a bona fide cargo of exosomes and in a soluble form. This new finding adds FAF1 to the list of proteins secreted by nonvesicular as well as exosomal routes.

### FAF1 positively regulates exosome number

**Fig. 3:**
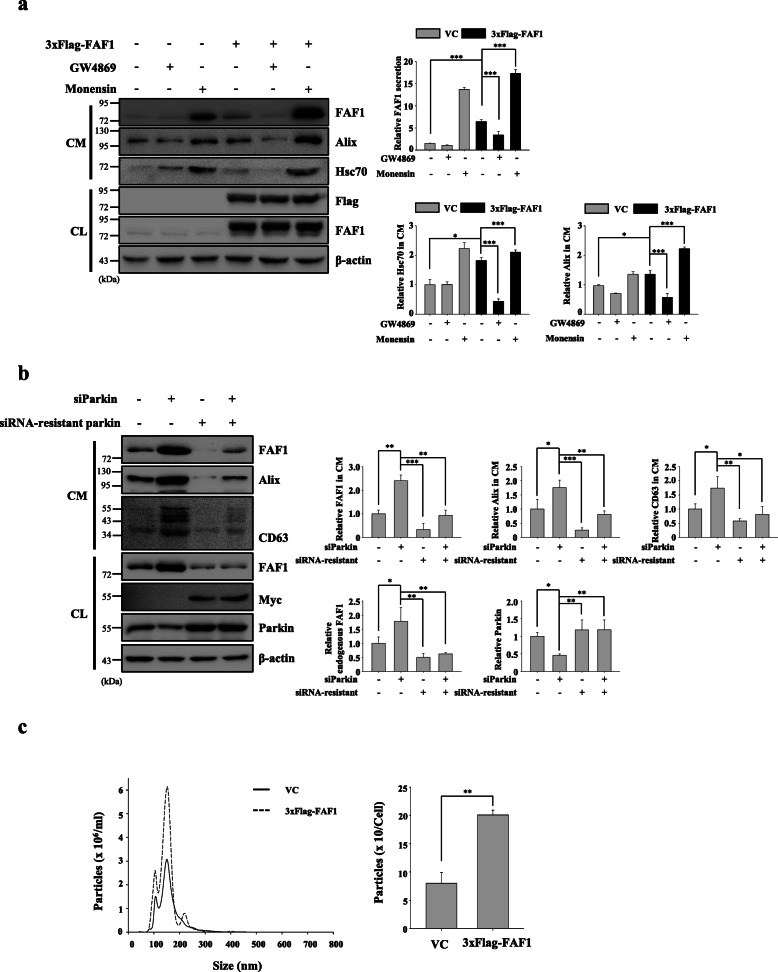
FAF1 positively controls exosome number. **a** SH-SY5Y cells were transfected with VC or 3xFlag-FAF1 plasmid. At 24 h after transfection, the culture medium was replaced with serum-free medium containing DMSO, GW4869 (10 μM), or monensin (10 μM), and the cells were cultured for 24 h. Left panel: CL and concentrated CM were analyzed by western blotting with the indicated antibodies. Right panel: The graph shows the results of densitometric analysis of FAF1, Alix, and Hsc70 immunoblots in the CM shown in the left panel (*n* = 3). All lanes were loaded with the same amount of total protein. **b** Cells were transfected with siRNA against parkin or siRNA-resistant parkin construct. At 24 h after transfection, the culture medium was replaced with serum-free medium, and the cells were cultured for 24 h. CL and concentrated CM were analyzed by western blotting with the indicated antibodies. Left panel: Representative western blots. Right panel: The graphs show the results of densitometric analysis of FAF1, Alix, and CD63 in CM, FAF1 and parkin in CL normalized to β-actin (*n* = 3). **c** Cells plated on 150 mm dishes were transfected with VC or 3xFlag-FAF1 plasmid. At 24 h after transfection, the culture medium was replaced with exosome-depleted medium, and the cells were cultured for 48 h. Then, exosomes were isolated from the CM with ExoQuick-TC. Left panel: Distribution profile of vesicles isolated by ExoQuick-TC. The blue line indicates exosomes from VC-transfected cells, and the red line indicates exosomes from 3xFlag-FAF1-transfected cells. Right panel: Exosome numbers were normalized to the final cell number (*n* = 3). The data are expressed as the mean ± S.D. of three independent experiments. Statistical comparisons were performed using ANOVA followed by Tukey’s HSD post hoc analysis **(a** and **b)** and Student’s t-test **(c)**. ^*^P < 0.05, ^**^P < 0.01, ^***^P < 0.001, and n.s. = not significant

The exosomal cargos such as Hsp20, Hsp90, and STAT3 increase exosome number [43–45]. Here, we examined whether FAF1 also participates in regulating exosome number. The exosome markers Hsc70, Alix, and CD63 were increased by 2 ± 0.09-fold, 1.5 ± 0.13-fold, and 3.3 ± 0.22-fold, respectively, in the CM of FAF1-transfected SH-SY5Y cells compared with control cells (Fig. 3a, Additional file 7: Fig. S6a). Next, we further studied exosome number changes using siRNA-mediated depletion of parkin, a E3-ubiquitin ligase of FAF1. siParkin treatment elevated secretion as well as expression of FAF1 in SH-SY5Y cells. Moreover, transfection of siRNA-resistant parkin construct reverted the increased secretion and expression of FAF1 by siParkin in SH-SY5Y cells. The expression levels of Alix and CD63 in the CM of SH-SY5Y cells in which parkin had been depleted were elevated by 1.75 ± 0.26- and 1.73 ± 0.40-fold, respectively (Fig. 3b). Collectively, these data imply that FAF1 positively controls exosome number. For the direct quantification of exosome number, nanoparticle tracking analysis was applied. The vesicle size distribution profile showing a diverse range of sizes showed that exosomes were present predominantly (Fig. 3c). The exosome numbers were normalized by cell number (Additional file 7: Fig. S6b). The exosomes of FAF1-transfected cells were increased by 2.5-fold compared to those of control cells. Hence, these data robustly demonstrate that FAF1 augments exosome number. In addition, GW4869 (an exosome release inhibitor) interfered with the ability of FAF1 to increase exosome number, while monensin (an exosome release promoter) enhanced this ability, implying that FAF1 functions upstream of the exosome release process (Fig. 3a) [46, 47].

### Secreted FAF1 is transmitted to adjacent cells

**Fig. 4:**
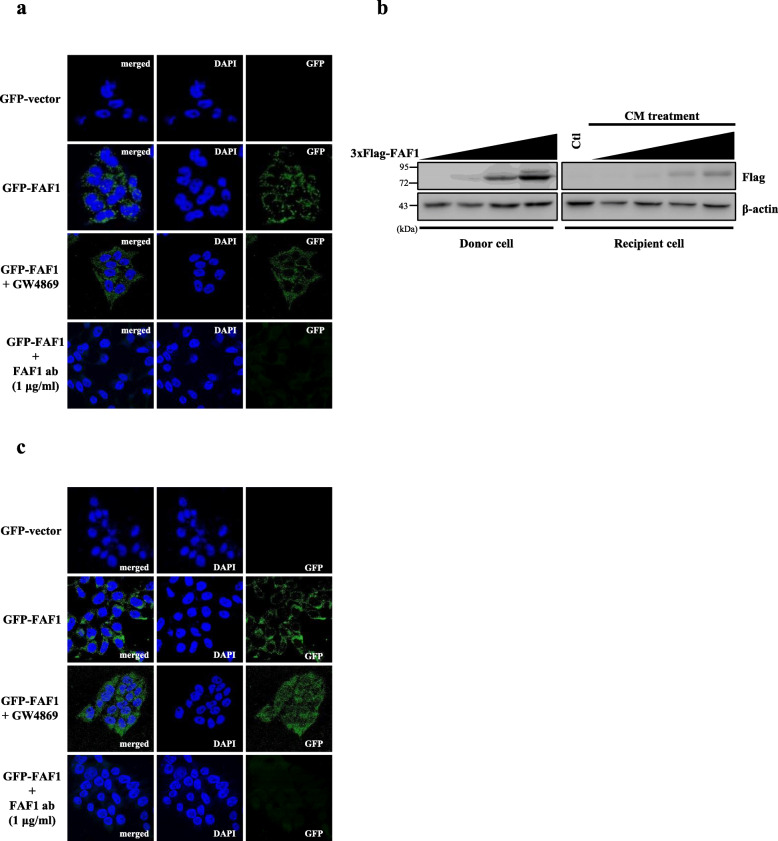
Secreted FAF1 is transmitted to neighboring cells. **a** Donor cells were transfected with GFP-vector or GFP-FAF1 plasmid. At 24 h after transfection, the cultured medium was replaced with serum-free medium containing DMSO or GW4869 (10 μM) and the cells were cultured for 24 h. FAF1 was immunodepleted from CM with anti-FAF1 monoclonal antibody (1 μg/ml). Concentrated CM was applied to recipient cells for 24 h. After the CM treatment, the nuclei of the recipient cells were stained using DAPI and analyzed by confocal microscopy. **b** Donor cells were transfected with VC or 3xFlag-FAF1 plasmid. At 24 h after transfection, the culture medium was replaced with serum-free medium, and the cells were cultured for 24 h. Concentrated CM was applied to recipient cells for 24 h. After CM treatment, CLs were analyzed by western blotting with the indicated antibodies. **c** Donor cells (insert) transfected with GFP-vector or GFP-FAF1 were coincubated with nontransfected recipient cells (lower compartment) in serum-free medium containing DMSO, GW4869 (10 μM), or anti-FAF1 monoclonal antibody (1 μg/ml) for 24 h. Then, the nuclei of the recipient cells were stained using DAPI and analyzed by confocal microscopy

We wondered whether extracellular FAF1 is internalized by neighboring cells. Donor cells were transfected with GFP or GFP-FAF1 plasmid for 24 h. Subsequently, the CM, which contained GFP or GFP-FAF1, was concentrated, and nontransfected recipient cells were treated with the concentrated CM for 24 h. Confocal microscopic images showed the presence of GFP-FAF1 in the cytoplasm of recipient cells, indicating that GFP-FAF1 had moved from donor cells to recipient cells. In contrast, GFP from the CM of donor cells was not detected in recipient cells, excluding the effect of tagging on transmission (Fig. 4a). Similarly, donor cells were transfected with 3xFlag-FAF1 plasmid as described above. Donor cell-derived FAF1 also moved into recipient cells, as shown by western blotting (Fig. 4b), corroborating the immunofluorescence results in Fig. 4a. In conclusion, these data consistently demonstrate that secreted FAF1 can be internalized by neighboring cells.

To further validate the cell-to-cell transfer of FAF1, an in vitro coculture system in which donor cells expressing GFP-FAF1 were incubated in upper transwell inserts while nontransfected recipient cells were incubated in lower compartments was used. Consistent with the above data, confocal microscopy of recipient cells revealed the presence of GFP-FAF1, indicating that GFP-FAF1 moved from cells in the upper transwell inserts to cells in the lower compartments (Fig. 4c). Consequently, these results show that extracellular FAF1 was transferred to neighboring cells without cell-to-cell contact.

We investigated the type of extracellular FAF1 that moves into recipient cells. To this end, donor cells were treated with GW4869, an exosome release inhibitor, after which recipient cells were analyzed by confocal microscopy. GW4869 failed to inhibit FAF1 transmission to recipient cells. To eliminate vesicle-free FAF1, CM of donor cells was immunodepleted with anti-FAF1 antibody and administered to recipient cells. Treatment with CM in which FAF1 was immunodepleted completely blocked FAF1 transmission to recipient cells, indicating that FAF1 transmission predominantly occurred through the transmission of vesicle-free FAF1 (Fig. 4a and c). These results are consistent with the above data (Fig. 2g and h) showing that most FAF1 is secreted via the nonvesicular pathway.

### Transmitted FAF1 in adjacent cells retains its death-promoting function

**Fig. 5:**
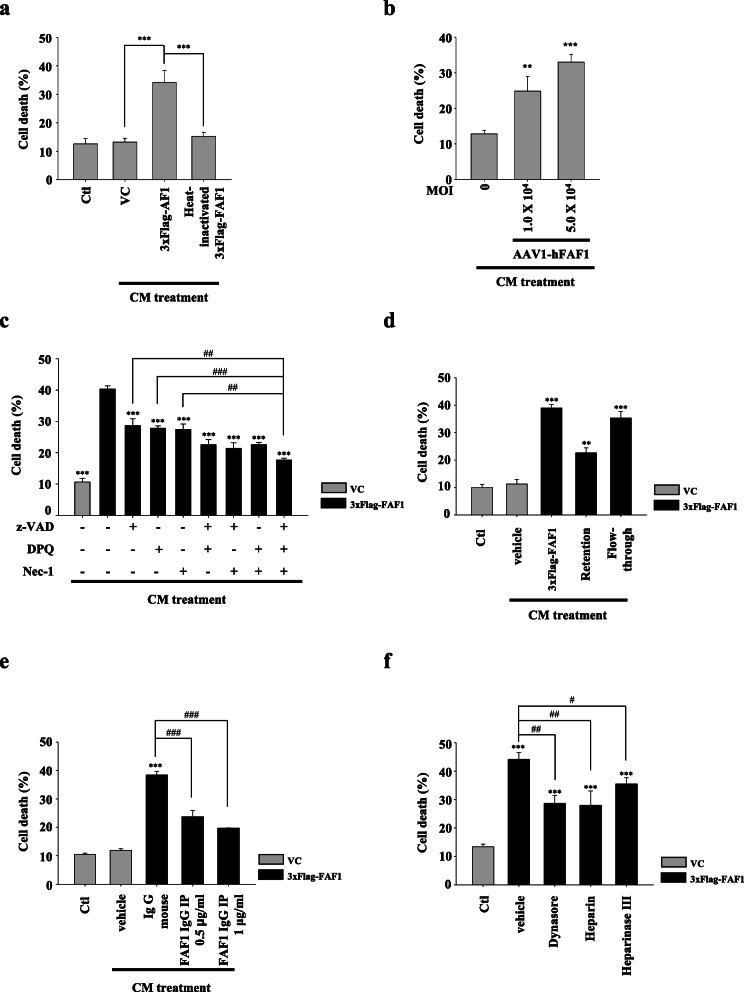
Transferred FAF1 in adjacent cells maintains its death function. Donor cells were transfected with VC or 3xFlag-FAF1 plasmid. At 24 h after transfection, the culture medium was replaced with serum-free medium, and the cells were cultured for 24 h. **a** The CM was applied to recipient cells for 48 h. Heat-inactivated CM was boiled for 10 min. Cell death was determined by measuring PI uptake using a flow cytometer. **b** Rat donor primary neuronal cells were transduced with AAV1-hFAF1 viral vectors. At 3 days after transduction, the culture medium was replaced with serum-free neurobasal medium for 48 h. The CM was applied to recipient primary neuronal cells for 48 h. Cell death was determined by measuring PI uptake using a flow cytometer. **c** The CM from vector-transfected cells or FAF1-transfected cells containing vehicle (DMSO), z-VAD-fmk (100 μM), DPQ (30 μM), or Nec-1 (50 μM) for 48 h was applied to recipient cells. **d** After the CM was fractionated into the retained fraction (exosomal FAF1) and flow-through (vesicle-free FAF1), each CM fraction was applied to recipient cells for 48 h. **e** FAF1 was immunodepleted from CM with anti-FAF1 monoclonal antibody, and the CM was applied to recipient cells for 48 h. **f** After the recipient cells were pretreated with Dynasore (80 μM), heparin (200 μg/ml), or heparinase III (0.01 IU/ml) for 24 h, CM was applied to recipient cells for 48 h. Cell death was determined by measuring PI uptake using a flow cytometer. Data are expressed as the mean ± S.D. of three independent experiments (*n* = 3). Statistical comparisons were performed using ANOVA followed by Tukey’s HSD post hoc analysis. ^**^P < 0.01, ^***^P < 0.001, ^#^*P* < 0.05, ^##^*P* < 0.01, and ^###^*P* < 0.001

The physiological functions of proteins transferred from cell to cell often manifest in the recipient cells [48–50]. Therefore, we asked whether FAF1 maintains its potential to kill recipient cells after transfer to a recipient cell. To address this, flow cytometric analysis was performed using PI staining. The CM from FAF1-transfected cells killed recipient SH-SY5Y cells, but heat-inactivated CM did not (Fig. 5a, Additional file 8: Fig. S7). Furthermore, extracellular FAF1 killed recipient rat primary neuronal cells in a dose-dependent manner, indicating that extracellular FAF1 retained its cell death potential in neighboring cells (Fig. 5b).

Then, the type of cell death caused by the CM of FAF1-transfected cells was examined. Caspase-3 activation was detected when SH-SY5Y cells were treated with the apoptosis inducer TNFα plus CHX (TC) for 6 h, demonstrating that the apoptotic machinery was intact in SH-SY5Y cells. Treatment with the CM of FAF1-transfected cells also triggered caspase-3 activation. However, treatment of z-IETD-fmk, a capase-8 inhibitor, significantly blocked caspase-3 activation by treatment of CM from FAF1-transfected cells in recipient cells, indicating that apoptosis is involved in recipient cell death induced by the CM of FAF1-transfected cells (Additional file 9: Fig. S8). Next, we examined the occurrence of other types of cell death. To this end, CM-treated cells were administered the indicated combinations of z-VAD (a pan-caspase inhibitor in apoptosis), DPQ (a PARP1 inhibitor in parthanatos), and Nec-1 (a RIPK1 inhibitor in necroptosis), which are key players in cell death. Each compound partially protected cells against the CM, whereas triple treatment protected cells far better, even though this protection was not complete (Fig. 5c). These data suggest that extracellular FAF1 induces recipient cell death through factors other than apoptosis, necroptosis, and parthanatos. Further studies are needed to elucidate the full molecular mechanisms underlying this cell death.

We wondered which type of extracellular FAF1 elicits recipient cell death. To address this, size-based filter centrifugation and immunodepletion were performed to distinguish between vesicle-free FAF1 (flow-through fraction) and exosomal FAF1 (retained fraction). The treatment of recipient cells with the flow-through fraction of the CM elicited striking cell death (Fig. 5d). In contrast, immunodepletion of FAF1 from FAF1-transfected CM abolished the majority of its toxicity to recipient cells (Fig. 5e). These data imply that CM-induced cell death is predominantly due to vesicle-free FAF1. Furthermore, the treatment of recipient cells with the retained fraction of the CM had modest cytotoxic effects on recipient cells (Fig. 5d), indicating the minor role of exosomal FAF1 in eliciting cell death. Collectively, these data revealed that vesicle-free FAF1 as well as exosomal FAF1, albeit to a lesser extent, retain FAF1 function to trigger cell death.

Certain pathogenic proteins such as tau and α-synuclein induce cell death via their internalization using heparan sulfate proteoglycan (HSPG)-mediated pinocytosis, a type of fluid-phase endocytosis [51]. Therefore, we examined whether extracellular FAF1 is also internalized via HSPG-mediated pinocytosis. To address this, recipient cells were pretreated with the HSPG inhibitors heparin and heparinase III for 24 h, followed by the application of CM from donor cells for 48 h. These inhibitors partially protected cells against the CM, indicating that secreted FAF1 is transmitted to recipient cells via HSPG-mediated pinocytosis, at least in part (Fig. 5f). Furthermore, Dynasore (a dynamin 1 inhibitor) reduced CM-induced cell death, suggesting that secreted FAF1 is also internalized by neighboring cells via clathrin-mediated endocytosis. Collectively, these results show that extracellular FAF1 is transferred to adjacent cells via pinocytosis as well as clathrin-mediated endocytosis.

## Discussion

Although FAF1 is known as an intracellular protein, this study reports that FAF1 is secreted into the extracellular space via an ER/Golgi-independent pathway. Moreover, FAF1 was found to be secreted through both vesicular and nonvesicular pathways. When these two types of FAF1 secretion were examined quantitatively, nonvesicular secretion predominated over vesicular secretion. Regarding vesicular secretion, FAF1 is transferred as a genuine cargo located in the lumen of exosomes and augments the number of exosomes. Furthermore, extracellular FAF1 can execute the death of neighboring cells through cell-to-cell transmission, implying that the transferred form of FAF1 retains the potential to trigger the death process.

The extracellular secretion of key proteins implicated in neurodegenerative diseases, including α-synuclein, tau, and prion, via both exosomal and nonvesicular pathways has been reported [18, 41, 42, 52]. Such secretion through dual pathways would accelerate their spread. Because FAF1 has been implicated in PD and is present in plasma and cerebrospinal fluid, PD progression would also be accelerated through cell-to-cell propagation of FAF1. Furthermore, vesicle-free forms of FAF1 predominated over the exosomal cargo form of FAF1 and was shown to play a prevalent role in triggering cell death, similar to α-synuclein [40]. Thus, neutralizing antibodies against soluble FAF1 could serve as an attractive neuroprotective therapeutic approach.

The regulation of exosome number is important because of its positive correlation with the severity of neurodegenerative diseases such as PD and Alzheimer’s disease [53]. Proteins involved in exosome biogenesis play regulatory roles in the secretory process at several positions. In particular, hepatocyte growth factor-regulated tyrosine kinase substrate (Hrs), a member of endosomal sorting complex required for transport (ESCRT), recruits monoubiquitinated proteins to ESCRT to accelerate exosome biogenesis [54]. Given that FAF1 also has ubiquitin-homologous domains, FAF1 could recruit monoubiquitinated cargo proteins to ESCRT to drive exosome biogenesis [29]. Alternatively, PD suppressors regulate exosome number by downregulating proteins involved in exosome biogenesis. For instance, parkin reduces exosome number through ubiquitin-mediated deactivation of Rab7, which augments exosome number in HEK293 cells [55]. Consistently, this study also showed that parkin depletion upregulated exosome secretion in SH-SY5Y cells. Considering that FAF1 is a substrate of parkin, a concomitant increase in FAF1 would have helped increase the exosome number. Therefore, it would be worthwhile to use FAF1 to facilitate exosome-mediated delivery of therapeutic protein cargos.

Pathogenic proteins in neurodegenerative disease are internalized by endocytosis, including pinocytosis and receptor-mediated endocytosis [56]. Like α-synuclein, tau, and prions, FAF1 is internalized by adjacent cells via HSPG-mediated pinocytosis [51]. Alternatively, some vesicle-free forms of proteins are also internalized via their specific receptors. For example, extracellular α-synuclein is internalized through its receptors, lymphocyte-activation gene 3 and dopamine transporter, and tau is internalized via CX3CR1 [57–59]. However, specific receptor(s) for FAF1 have not yet been discovered. The identification of FAF1 receptor(s) would be useful because it would open a new window for therapeutic opportunities in neurodegenerative diseases.

In PD pathogenesis, a feed-forward loop between stressor proteins in PD is formed. Specifically, pathologic α-synuclein drives poly (ADP)-ribose (PAR) polymerase-1 (PARP-1), and PAR accelerates the conversion of α-synuclein to its toxic forms, facilitating pathologic α-synuclein transmission [60]. Given that FAF1 also activates PARP-1 and FAF1 expression is elevated upon oxidative stress [34], a feed-forward loop between FAF1 and PARP-1 might exist. Moreover, it would be worthwhile to study the presence of molecular interplay between FAF1, α-synuclein, and PARP-1.

FAF1 is a tumor suppressor and metastasis inhibitor whose expression is downregulated in various cancers [31, 61]. Because extracellular FAF1 retains its death function, FAF1 delivery via an extracellular route is a plausible therapeutic strategy. In particular, vesicle-free FAF1 could be transferred directly to neighboring cells. Therefore, intratumoral administration of FAF1 is worth investigating. Furthermore, FAF1 would be a suitable therapeutic candidate for exosome-mediated delivery because of the compatibility between FAF1 as a genuine cargo and exosomes. The positive regulatory function of FAF1 in exosome number shows FAF1 as an even better anticancer therapeutic candidate.

FAF1 participates in proteostatic regulation of other proteins including Hsp70, Aur-A and α-synuclein [27, 61]. However, study on the proteostatic regulation for FAF1 itself is relatively limited except for the negative regulation by parkin-mediated ubiquitin-proteasome system. Furthermore, only NF-κB is known as transcription factor for FAF1 [62]. Interestingly, FAF1 inhibits NF-κB activation by disrupting IKK complex formation as well as interfering nuclear translocation of RelA (p65) [25, 26], demonstrating the presence of negative feedback loop exists between FAF1 and NF-κB. Further studies on upstream regulatory mechanisms of FAF1 are needed.

## Conclusions

This study reports for the first time that FAF1 is secreted to the extracellular space via an ER/Golgi-independent pathway. In the extracellular space, FAF1 is transferred through nonvesicular as well as exosomal pathways. Extracellular FAF1 is transmitted to adjacent cells via clathrin-mediated endocytosis and pinocytosis. Transmitted FAF1 in neighboring cells retains its death function. In addition to the role of cytoplasmic FAF1 in promoting PD pathogenesis, this study presents a novel molecular mechanism of FAF1 in neurodegeneration, i.e., its cell-to-cell transmission. Our findings open a new window into interference with FAF1-mediated cell death via the inhibition of FAF1 secretion.

## Supplementary information

**Additional file 1.** FAF1 is detected in plasma and CSF according to the CSF Proteome Resource.

**Additional file 2 Figure S1**. FAF1 secretion is not an artificial effect. **a** Cells were transfected with VC or 3xFlag-FAF1 plasmid. At 24 h after transfection, cell death was determined by measuring propidium iodide uptake using a flow cytometer (*n* = 3). **b** Cells were transfected with VC or 3xFlag-FAF1 plasmid. At 24 h after transfection, the culture medium was replaced with serum-free medium, and the cells were cultured for 24 h. CL and concentrated CM were analyzed by western blotting with the indicated antibodies. **c** Cells were transfected with VC or HA-FAF1 plasmid. At 24 h after transfection, the culture medium was replaced with serum-free medium, and the cells were cultured for 24 h. CL and concentrated CM were analyzed by western blotting with the indicated antibodies. **d** Cells were transfected with 3xFlag-FAF1 plasmid. At 24 h after transfection, the culture medium was replaced with serum-free medium containing BFA (2 μg/ml) for 24 h. Subsequently, the recipient cells were stained using DAPI, GM130, and DCF-DA as indicated and analyzed by confocal microscopy. Statistical comparisons were performed using ANOVA followed by Tukey’s HSD post hoc analysis. ^***^P < 0.001.

**Additional file 3 Figure S2**. FAF1 is secreted from various cell lines. **a-f** MEF, HEK293, MCF-7, HeLa, PANC-1, and MIA PaCa-2 cells were transfected with VC or 3xFlag-FAF1 plasmid. At 24 h after transfection, the culture medium was replaced with serum-free medium, and the cells were cultured for 24 h. Concentrated CM was analyzed by western blotting with the indicated antibodies.

**Additional file 4 Figure S3**. PD stressors are positive regulators of FAF1 secretion. Cells were transfected with VC or 3xFlag-FAF1 plus α-syn plasmid. At 24 h after transfection, the culture medium was replaced with serum-free medium containing DMSO (vehicle), MPP^+^ (1 mM), H_2_O_2_ (100 μM), or Baf A1 (50 nM), and the cells were cultured for 24 h. Cell death was determined by measuring PI uptake using a flow cytometer (n = 3).

**Additional file 5 Figure S4.** FAF1 lacks signal peptides. Signal P outputs for secretogranin-1 and FAF1. Left panel: secretogranin-1, a member of secretory vesicle, contains a typical signal peptide. Right panel: FAF1 has no signal peptide.

**Additional file 6 Figure S5.** FAF1 is secreted in exosomes in various cell lines. **a-h** SH-SY5Y, MEF, HEK293, RAW264.7, HeLa, PANC-1, Mia-PaCa2, and MCF-7 cells plated on 150 mm diameter dishes were transfected with VC or 3xFlag-FAF1 plasmid. At 24 h after transfection, the culture medium was replaced with exosome-depleted medium, and the cells were cultured for 48 h. The exosomes were isolated from CM with ExoQuick-TC. Exosomes were analyzed by western blotting with the indicated antibodies.

**Additional file 7 Figure S6.** FAF1 upregulation increases exosome number. SH-SY5Y cells plated on 150 mm dishes were transfected with VC or 3xFlag-FAF1 plasmid. At 24 h after transfection, the culture medium was replaced with exosome-depleted medium, and the cells were cultured for 48 h. **a** After exosomes were isolated from the CM of each group of cells with ExoQuick-TC, CL and isolated EXOs were analyzed by western blotting with the indicated antibodies. **b** Final cell numbers were determined using a Muse analyzer. Statistical comparisons were evaluated using ANOVA followed by Tukey’s HSD post hoc analysis. ^***^P < 0.001, and n.s. = not significant.

**Additional file 8 Figure S7.** Recipient cell death was not changed in response to CMs of SH-SY5Y cells transtected with FAF1 with different tags. Donor cells were transfected with VC, no-tagged FAF1, and HA-FAF1 plasmid. At 24 h after transfection, the culture medium was replaced with serum-free medium, and the cells were cultured for 24 h. The CM was applied to recipient cells for 48 h. Heat-inactivated CM was boiled for 10 min. Cell death was determined by measuring PI uptake using a flow cytometer. Statistical comparisons were evaluated using ANOVA followed by Tukey’s HSD post hoc analysis. ^**^P < 0.01 and ^***^P < 0.001.

**Additional file 9 Figure S8.** CM from FAF1-transtected cells induces neighboring cell death via caspase-3 activation. Donor cells were transfected with VC or 3xFlag-FAF1 plasmid. At 24 h after transfection, the culture medium was replaced with serum-free medium plus z-IET-fmk (20 μM), a caspase-8 inhibitor, and the cells were cultured for 24 h. After the CM after culture for 48 h or treatment with TNFα (50 ng/ml) plus CHX (20 μg/ml) for 6 h was applied to recipient cells, caspase-3 activity was analyzed using fluorometric assays. TC: TNFα + CHX. Statistical comparisons were evaluated using ANOVA followed by Tukey’s HSD post hoc analysis. ^*^P < 0.05, ^**^P < 0.01 and ^***^P < 0.001.

## Data Availability

All data generated or analyzed during this study are included in this published article.

## Abbreviations

FAF1: Fas-associated factor 1
CM: conditioned medium
