# Supplementary figures and images for: A novel function of FAF1, which induces dopaminergic neuronal death through cell-to-cell transmission
Source: Cell Commun Signal. 2020 Aug 24;18:133. doi: 10.1186/s12964-020-00632-8 (PMC7444258; doi:10.1186/s12964-020-00632-8)

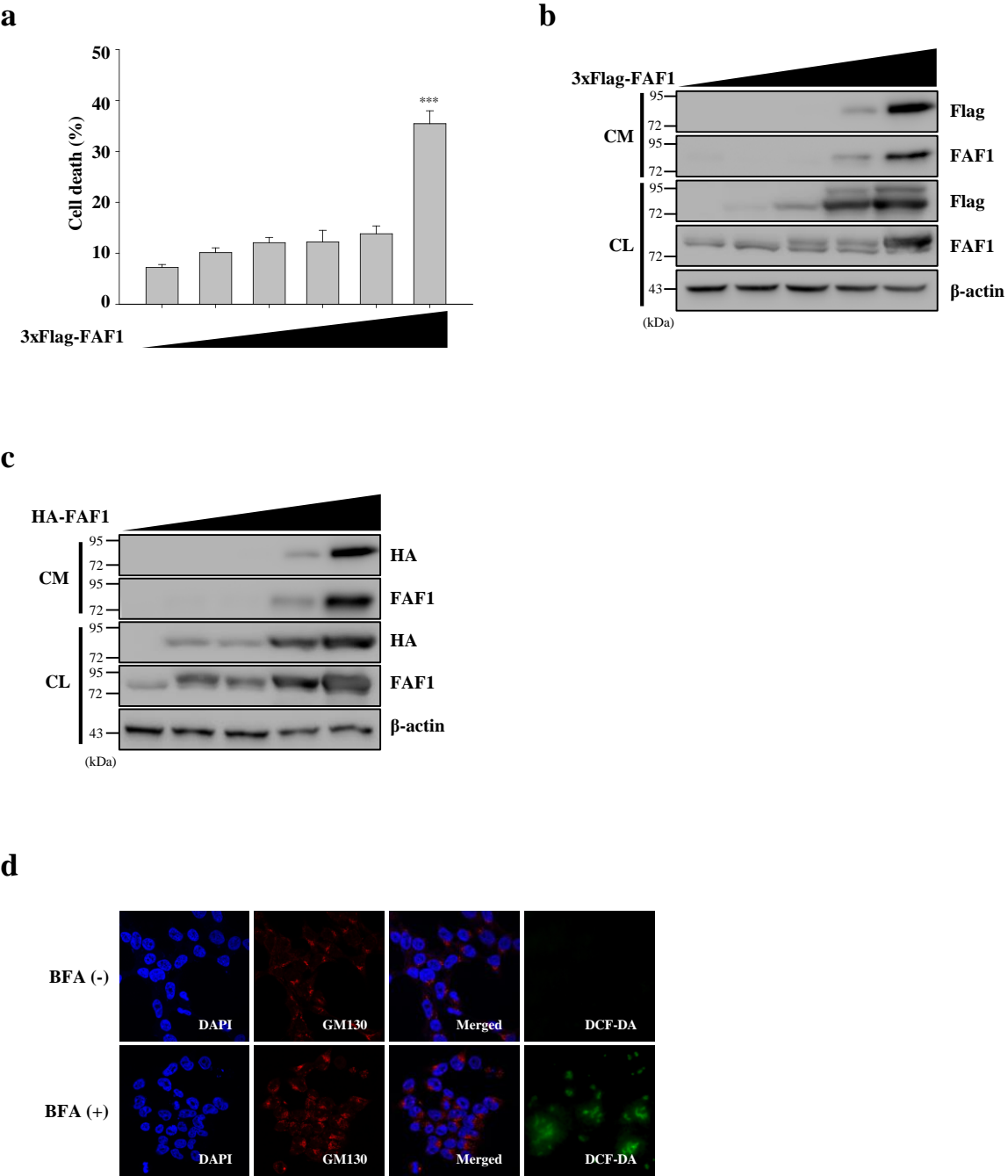

Supplement: Supplementary file 3 — Additional file 2 Figure S1. FAF1 secretion is not an artificial effect. a Cells were transfected with VC or 3xFlag-FAF1 plasmid. At 24 h after transfection, cell death was determined by measuring propidium iodide uptake using a flow cytometer (n = 3). b Cells were transfected with VC or 3xFlag-FAF1 plasmid. At 24 h after transfection, the culture medium was replaced with serum-free medium, and the cells were cultured for 24 h. CL and concentrated CM were analyzed by western blotting with the indicated antibodies. c Cells were transfected with VC or HA-FAF1 plasmid. At 24 h after transfection, the culture medium was replaced with serum-free medium, and the cells were cultured for 24 h. CL and concentrated CM were analyzed by western blotting with the indicated antibodies. d Cells were transfected with 3xFlag-FAF1 plasmid. At 24 h after transfection, the culture medium was replaced with serum-free medium containing BFA (2 μg/ml) for 24 h. Subsequently, the recipient cells were stained using DAPI, GM130, and DCF-DA as indicated and analyzed by confocal microscopy. Statistical comparisons were performed using ANOVA followed by Tukey’s HSD post hoc analysis. ***P < 0.001. [file 12964_2020_632_MOESM3_ESM.pdf]

**a**

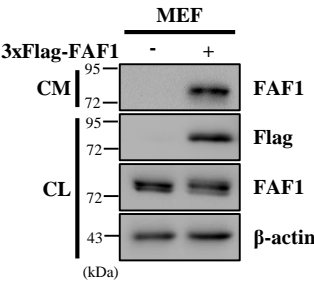

**b**

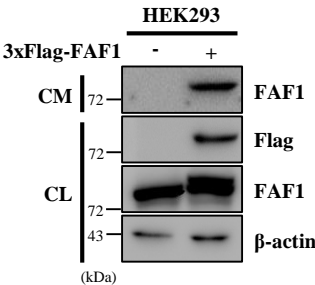

**c**

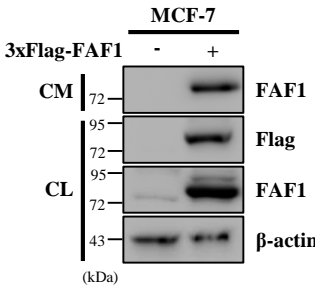

**d**

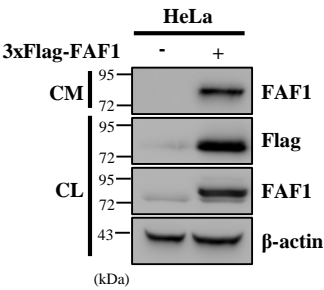

**e**

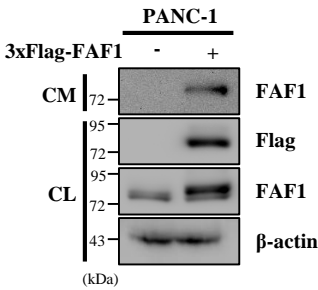

**f**

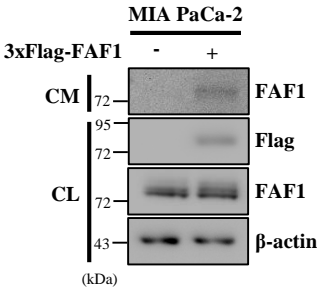

Supplement: Supplementary file 4 — Additional file 3 Figure S2. FAF1 is secreted from various cell lines. a-f MEF, HEK293, MCF-7, HeLa, PANC-1, and MIA PaCa-2 cells were transfected with VC or 3xFlag-FAF1 plasmid. At 24 h after transfection, the culture medium was replaced with serum-free medium, and the cells were cultured for 24 h. Concentrated CM was analyzed by western blotting with the indicated antibodies. [file 12964_2020_632_MOESM4_ESM.pdf]

## Additional file 4: Figure S3

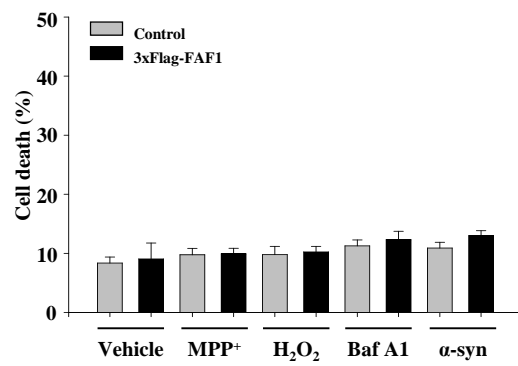

Supplement: Supplementary file 5 — Additional file 4 Figure S3. PD stressors are positive regulators of FAF1 secretion. Cells were transfected with VC or 3xFlag-FAF1 plus α-syn plasmid. At 24 h after transfection, the culture medium was replaced with serum-free medium containing DMSO (vehicle), MPP+ (1 mM), H2O2 (100 μM), or Baf A1 (50 nM), and the cells were cultured for 24 h. Cell death was determined by measuring PI uptake using a flow cytometer (n = 3). [file 12964_2020_632_MOESM5_ESM.pdf]

Additional file 5: Figure S4

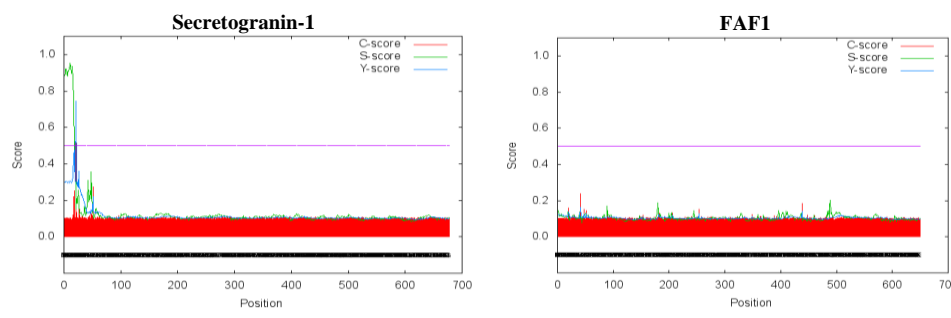

Supplement: Supplementary file 6 — Additional file 5 Figure S4. FAF1 lacks signal peptides. Signal P outputs for secretogranin-1 and FAF1. Left panel: secretogranin-1, a member of secretory vesicle, contains a typical signal peptide. Right panel: FAF1 has no signal peptide. [file 12964_2020_632_MOESM6_ESM.pdf]

Additional file 6: Figure S5

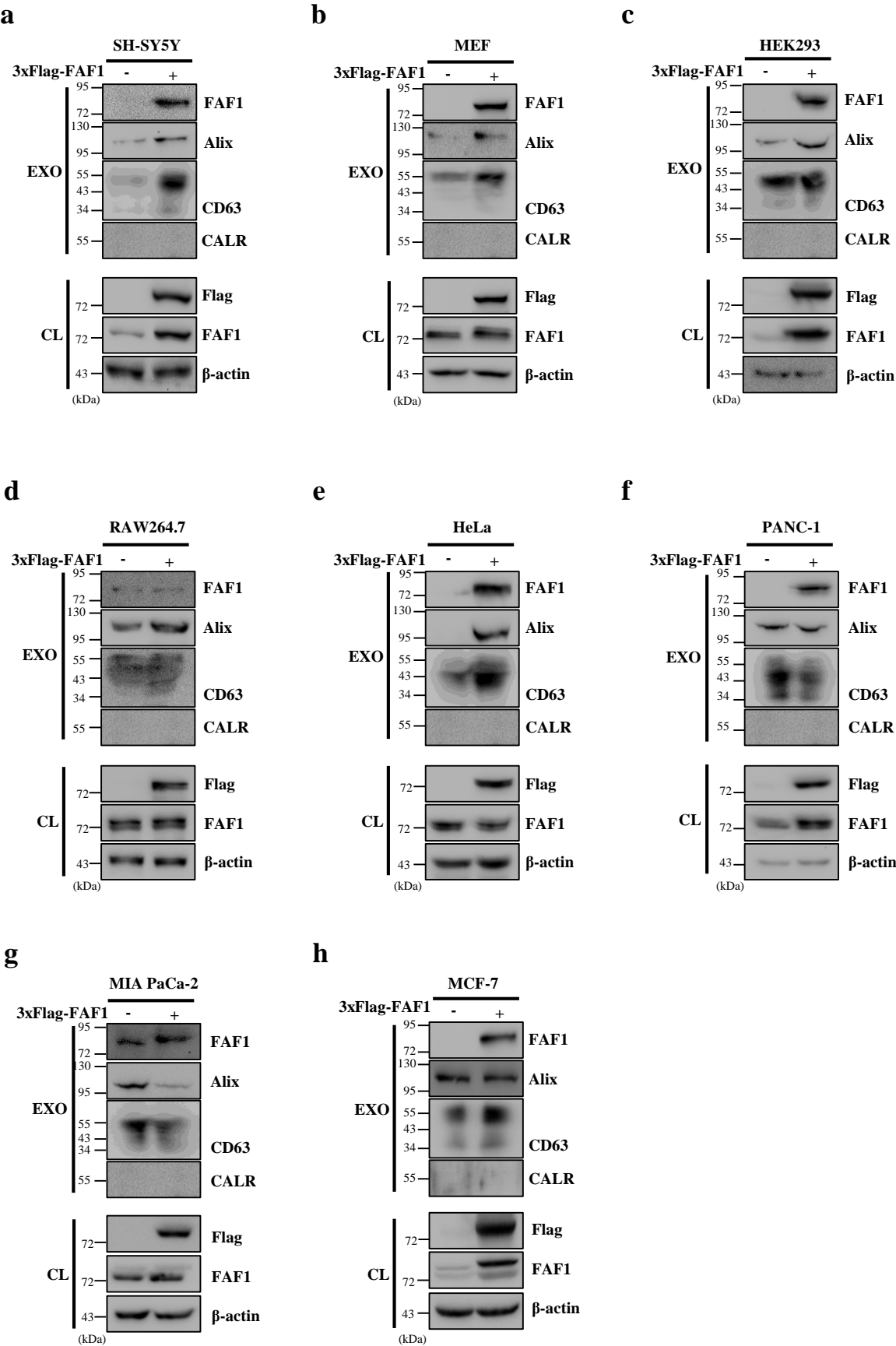

Supplement: Supplementary file 7 — Additional file 6 Figure S5. FAF1 is secreted in exosomes in various cell lines. a-h SH-SY5Y, MEF, HEK293, RAW264.7, HeLa, PANC-1, Mia-PaCa2, and MCF-7 cells plated on 150 mm diameter dishes were transfected with VC or 3xFlag-FAF1 plasmid. At 24 h after transfection, the culture medium was replaced with exosome-depleted medium, and the cells were cultured for 48 h. The exosomes were isolated from CM with ExoQuick-TC. Exosomes were analyzed by western blotting with the indicated antibodies. [file 12964_2020_632_MOESM7_ESM.pdf]

**a**

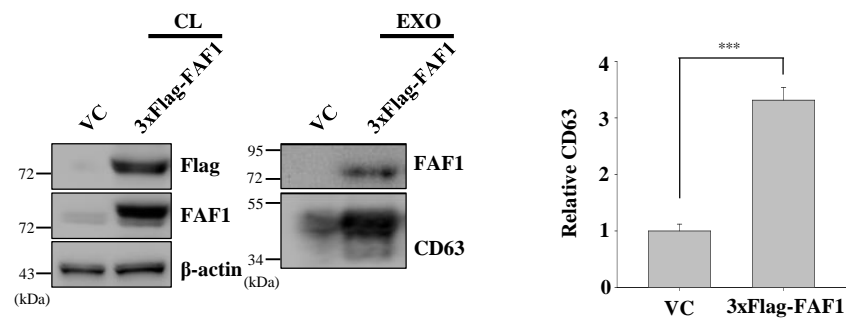

**b**

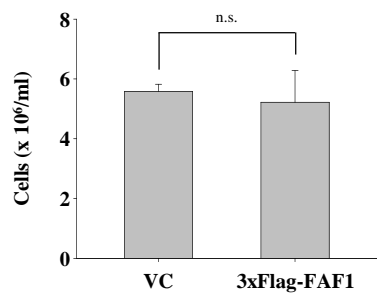

Supplement: Supplementary file 8 — Additional file 7 Figure S6. FAF1 upregulation increases exosome number. SH-SY5Y cells plated on 150 mm dishes were transfected with VC or 3xFlag-FAF1 plasmid. At 24 h after transfection, the culture medium was replaced with exosome-depleted medium, and the cells were cultured for 48 h. a After exosomes were isolated from the CM of each group of cells with ExoQuick-TC, CL and isolated EXOs were analyzed by western blotting with the indicated antibodies. b Final cell numbers were determined using a Muse analyzer. Statistical comparisons were evaluated using ANOVA followed by Tukey’s HSD post hoc analysis. ***P < 0.001, and n.s. = not significant. [file 12964_2020_632_MOESM8_ESM.pdf]

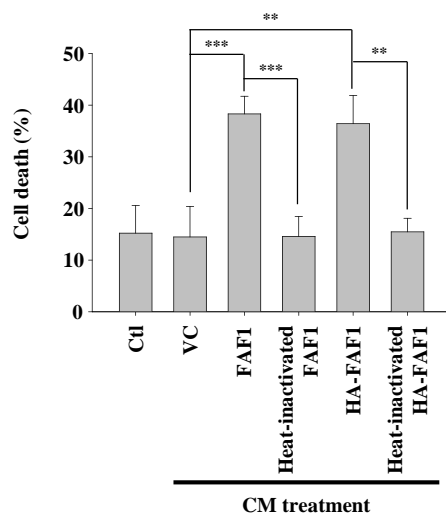

Supplement: Supplementary file 9 — Additional file 8 Figure S7. Recipient cell death was not changed in response to CMs of SH-SY5Y cells transtected with FAF1 with different tags. Donor cells were transfected with VC, no-tagged FAF1, and HA-FAF1 plasmid. At 24 h after transfection, the culture medium was replaced with serum-free medium, and the cells were cultured for 24 h. The CM was applied to recipient cells for 48 h. Heat-inactivated CM was boiled for 10 min. Cell death was determined by measuring PI uptake using a flow cytometer. Statistical comparisons were evaluated using ANOVA followed by Tukey’s HSD post hoc analysis. **P < 0.01 and ***P < 0.001. [file 12964_2020_632_MOESM9_ESM.pdf]

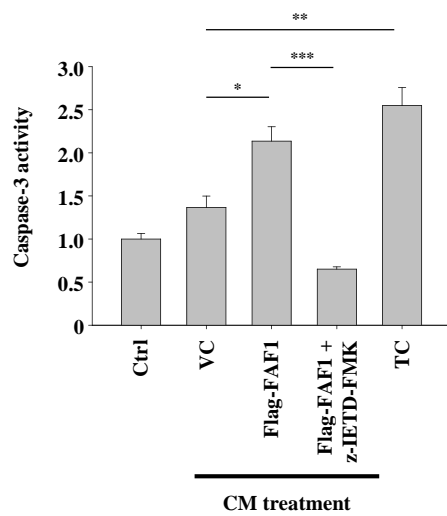

Supplement: Supplementary file 10 — Additional file 9 Figure S8. CM from FAF1-transtected cells induces neighboring cell death via caspase-3 activation. Donor cells were transfected with VC or 3xFlag-FAF1 plasmid. At 24 h after transfection, the culture medium was replaced with serum-free medium plus z-IET-fmk (20 μM), a caspase-8 inhibitor, and the cells were cultured for 24 h. After the CM after culture for 48 h or treatment with TNFα (50 ng/ml) plus CHX (20 μg/ml) for 6 h was applied to recipient cells, caspase-3 activity was analyzed using fluorometric assays. TC: TNFα + CHX. Statistical comparisons were evaluated using ANOVA followed by Tukey’s HSD post hoc analysis. *P < 0.05, **P < 0.01 and ***P < 0.001. [file 12964_2020_632_MOESM10_ESM.pdf]
